# Supplementary material for: A UMLS-based spell checker for natural language processing in vaccine safety
Source: BMC Med Inform Decis Mak. 2007 Feb 12;7:3. doi: 10.1186/1472-6947-7-3 (PMC1805499; doi:10.1186/1472-6947-7-3)
Supplement: Additional file 13 — RAP application directory. Sets up directory for RAP (RDF application for PHP) source code [file 1472-6947-7-3-S13.gz › rap/doc/phpdoc/__filesource/fsource_default__infModelInfModelP.php.html]

xml version="1.0" encoding="iso-8859-1"?


File Source for InfModelP.php


# Source for file InfModelP.php

Documentation is available at InfModelP.php

```
1. <?PHP
2. // ----------------------------------------------------------------------------------
3. // InfModel Package
4. // ----------------------------------------------------------------------------------
5. //
6. // Description               : InfModel package
7. //
8. // History:
9. // 09-10-2004                : Initial version.
10. //
11. // Author: Daniel Westphal    <mail at d-westphal dot de>
12. //
13. // ----------------------------------------------------------------------------------

15. // Include InfModel classes

17. require_once( RDFAPI_INCLUDE_DIR . 'infmodel/InfRule.php');
18. require_once( RDFAPI_INCLUDE_DIR . 'infmodel/InfStatement.php');
19. require_once( RDFAPI_INCLUDE_DIR . 'infmodel/InfModel.php');
20. require_once( RDFAPI_INCLUDE_DIR . 'infmodel/InfModelB.php');
21. require_once( RDFAPI_INCLUDE_DIR . 'infmodel/InfModelF.php');
22. ?>
```

Documentation generated on Fri, 17 Dec 2004 16:15:24 +0100 by phpDocumentor 1.3.0RC3
